# Active Ageing: The Need to Address Sub-National Diversity. An Evidence-Based Approach for Italy

**DOI:** 10.3390/ijerph182413319

**Published:** 2021-12-17

**Authors:** Marina Zannella, Andrea Principi, Davide Lucantoni, Francesco Barbabella, Mirko Di Rosa, Antía Domínguez-Rodríguez, Marco Socci

**Affiliations:** 1 Centre for Socio-Economic Research on Ageing, National Institute of Health and Science on Ageing (IRCCS INRCA), 60124 Ancona, Italy; m.zannella@inrca.it (M.Z.); a.principi@inrca.it (A.P.); f.barbabella@inrca.it (F.B.); m.socci@inrca.it (M.S.); 2 Unit of Geriatric Pharmacoepidemiology and Biostatistics, National Institute of Health and Science on Ageing (IRCCS INRCA), 60124 Ancona, Italy; m.dirosa@inrca.it; 3 Social Determinants of Health and Demographic Change-Opik (UPV/EHU) and ESOMI—Faculty of Sociology, University of a Coruña (UDC), 15008 Galicia, Spain; a.drodriguez@udc.es

**Keywords:** active ageing, Active Ageing Index, demographic dynamics, regional studies, gender, evidence-based policy, equity, sustainability

## Abstract

While active ageing has emerged as a main strategy to address the challenges of population ageing in Europe, recent research has stressed the need to increase knowledge on within-country differences to promote active ageing through appropriate policy responses. This article draws on the Active Ageing Index (AAI) to capture recent trends in active ageing in Italy with a focus on sub-national diversity. To this end, we compute AAI breakdowns by region separately for men and women for four different years: 2007, 2009, 2012 and 2018. Then, we use linear regression to describe the geographical and sex-specific patterns of change in the AAI over the considered period. The results demonstrate the diversity of regional outcomes and trends in the active ageing of Italian men and women, indicating that the widening geographic gap deserves further consideration by national and regional authorities in designing and implementing active ageing policies. By showing the persistence of disparities in the value of the indicator to the disadvantage of women, results also suggest the need to further integrate both the gender dimension and the life-cycle perspective into active ageing strategies. This article provides an example of how the AAI can be used as a practical tool by policy makers to monitor active ageing trends and outcomes at the sub-national level, and to identify target areas that require further action.

## 1. Introduction

Population ageing is one of the demographic “megatrends” with continued and long-lasting impacts on sustainable development and individual lives [1,2]. Indeed, while increasing longevity is considered one of the greatest achievements of the 21st century, extending life length can imply both opportunities and challenges for contemporary and future societies. To enhance the positive aspects and reduce the risks associated with longer lives, “active ageing” has been proposed as a “process of optimizing opportunities for health, participation and security in order to enhance quality of life as people age” [3]. The concept of active ageing not only indicates the individual processes of surviving old age in good health and actively participating in society, but also refers to social processes supported by sound policies and programmatic interventions that can help societies age well [4]. Active ageing is, therefore, a ‘win-win’ strategy that benefits both older individuals, in terms of improved health and well-being, and societies at large [5]. However, the conceptualisation and operationalisation of active ageing has some risks, one of the most common being to interpret it in a unidimensional approach using exclusively an economic/productive framework [6] or a strongly health-oriented perspective [7]. Other common risks, which can also occur in multidimensional interpretations of active ageing, is not considering the heterogeneity of personal preferences and aspirations of older individuals as well as adopting a top-down approach in active ageing policy development [8]. Nevertheless, in recent decades, policymakers and scholars have taken important steps by leading several international, national and regional initiatives to clarify the multidimensional nature of active ageing and adopting coherent and integrated policies to address the needs, attitudes and preferences of ageing individuals [9].

Although population ageing is a global issue, the ageing process is more advanced in Europe than in other parts of the world and demographic projections suggest that the ageing of the European population will become even faster in the near future [10]. Against this background, ageing emerged as a main European policy issue in the early 1990s and, since then, the European Union (EU) has increased its efforts to adopt active ageing as an explicit and long-term policy goal [11]. The beginning of the new millennium marked the transition to an era of active ageing mainstreaming: several policy initiatives took place at the international level as well as at the national and sub-national level, especially in Europe, with the aim of adapting welfare arrangements to the active ageing paradigm. However, despite much positive political rhetoric, it is evident that there is still considerable uncertainty about what active ageing should mean in practice [12]. In fact, within most national governments, including Italy, the policy debate on ageing issues has been focused on pension and social security reforms and not on effective strategies for activating older people [13]. The role of the public sector in redistributing resources across generations has received much attention and scholars have mainly studied aspects related to fiscal sustainability and intergenerational equity [14]. Solidarity between generations at all levels—family, community, state—has been universally identified as being of paramount importance in a context of significant demographic and socio-economic change [15]. However, it has been explicitly recognised that intergenerational solidarity, or the generational contract, relies heavily on the existence of a gender contract [16], which is defined as the explicit and implicit rules governing gender relations and the assignment of different work, values and responsibilities to men and women [17]. Nevertheless, ageing strategies do not yet take into proper account the gendered nature of ageing and earlier life course events [12].

Developing the full potential of active ageing is of great relevance for Italy which, demographically speaking, is the second oldest country in the world and the oldest in Europe [1]. In 2020, 23.2% of the Italian population was 65 years and older, against the EU-28 average of 20.6%; while the corresponding figures for the population aged 80+ were 7.4% and 5.9% respectively (Eurostat data accessed 11 November 2021, https://ec.europa.eu/eurostat/data/database). The peak of ageing will hit Italy in 2045–50, when a share of almost 34% of the population will be 65 years and older [18]. Italy can therefore be considered a paradigmatic example of the need to develop effective policy responses to manage the ageing process, as the pace and intensity of demographic change in this country require structural adaptability and flexibility that must be achieved rapidly [19].

In this framework, this article aims to assess recent developments towards active ageing in Italy with a gender and territorial perspective. To this end, we rely on the Active Ageing Index (AAI): a tool developed to measure active ageing in a given geographical and socioeconomic context that can, therefore, provide an evidence-based guidance to promote active ageing through policies at regional/local level rather than only at the European and national level [20]. First, we estimate AAI breakdowns by region separately for men and women and present the related descriptive statistics. Then, we use linear regression to describe the geographical and sex-specific patterns of change of the AAI during the period considered.

The focus on gender and territorial dimensions is intrinsically relevant for the analysis of active ageing considering that, while active ageing depends on a variety of factors and characteristics affecting individuals, families and societies, two cross-cutting determinants of this process have been identified: culture and gender [3]. Respecting national and cultural diversity is, therefore, of paramount importance to achieve the full potential of active ageing [11]. Differences between northern and southern Europe have been noted in the forms of active participation undertaken in old age [21]. However, even at the national level there are often considerable cultural differences across the territory, as in the case of Italy where regional differences in participation as well as gender norms are pronounced [22]. In addition to these differences, Italy is an unequal and diverse country characterized by a remarkable North-South divide in terms of demographic, economic, social and environmental conditions [23], which demonstrates an urgent need of increasing the knowledge on regional diversity in active ageing [20], in order to support future policy making at the regional level in an innovative way (i.e., by employing the AAI).

The article is organized as follows. Section 2 discusses the relevance of the gender and territorial dimensions for active ageing in the Italian context and describes the motivation and the research questions of the paper. Section 3 provides methodological explanations of the AAI and of its applications for the purposes of this article. Section 4 shows the results, that are further discussed in Section 5. In Section 6 conclusions are drawn.

## 2. Background

From a demographic point of view, population ageing is the result of a process of declines in both mortality and fertility and the consequent shift in cohorts’ size. Europe moved into the ageing process in the 1960s when life expectancy at birth recorded significant increases, especially for women. The magnitude of these gains has been pronounced in Italy where life expectancy at birth increased by more than 11 years from 1960 to 2012 [24]. Similarly, the decline in fertility that took place in Europe after the post-war baby boom had greater effects in Italy than in other countries: a historic low Total Fertility Rate (TFR) of less than 1.2 children per woman was reached in the mid-1990s and lowest low fertility (lowest-low fertility is defined as TFR at or below 1.3) levels were recorded for over 25 years [25]. During the first decade of the new millennium, fertility rates have increased in almost all Member States. The magnitude of the increase has been greater in a number of European countries, especially those where the fall begun earliest, attenuating the ageing process. This is not the case in Italy, where the drop in fertility that followed the 2008 economic crisis has been pronounced [25]. Fertility differentials in Europe, and in particular differences between low fertility countries (i.e., fertility moderately below the replacement level of 2.1 children per woman) and lowest-low fertility countries, are at least partly explained by the character of family and gender equality, and, more generally, by differences in welfare state regimes and family policies [26]. The Italian welfare system belongs to the Mediterranean model, characterised by weak family support policies together the expectation of extended family solidarity and responsibility for care, including care for older people [27]. From the point of view of care policies, the Italian welfare system can be described as a mix of *familialism by default* and *supported familialism* and a low degree of *defamilization* (the term supported familialism is used to describe a context in which policies actively support women in assuming the main responsibilities for family care needs and encourage mothers to remain in the labour market by providing extended job protection for long periods of parental leave; whereas, the term familialism by default describes a situation in which the shift of intergenerational responsibilities from the State to families is accompanied by little or no policy and financial support. Finally, the term defamiliarisation is used to denote an active role of the state in providing assistance to dependent members of the population and, thus, lifting part of the intergenerational responsibilities from families), with the incidence of *familialism by default* being greater for the disabled and older members of the population than for children [28]. Substantial regional differences are observed in the use and availability of formal care at the disadvantage of the South. Indeed, gender asymmetries in the division of family care are considerable throughout the whole territory but the magnitude of these differences is greater in the South and Islands than in the rest of the country [29]. Differences in unpaid domestic work and family care are marked also across dual earner couples: the combination of paid and unpaid responsibilities results in less time available for women for personal care, social contacts and activities that contribute to create social capital [30] and can, therefore, positively influence individual well-being and the ageing process.

Considerable differences across the territory exist in relation of many other dimensions. For example, the quality of health services is higher in the North than in the Centre and especially in the South; moreover, GDP and average pension income are higher in the North. Individuals in the northern regions are also better off in relation to aspects of subjective well-being such as self-reported quality of life, family relations, social relations, trust, etc. [29]. Several policies that have important effects on the well-being of people and society are local, and local are, very often, the interactions between the policies themselves [31]. This is particularly true in Italy where a progressive growth in the autonomy and responsibility of local authorities took place in the last decades. Ageing issues are, therefore, currently distributed among different Ministries and government levels with Regional Governments being responsible for most active ageing issues including active labour market policies, training policies, the local organization of the National Health Service and the coordination of social services and municipalities providing for social services [32]. The territory is one of the main keys to understanding inequalities in Italy: in the territory, inequalities interact with each other, accentuating conditions of disadvantage or reinforcing positions of advantage [33].

A limited number of studies have analyzed active ageing at the subnational level in Italy, showing the existence of considerable gender and geographical differences [34,35,36]. Based on these studies and in the light of the considerations above, this article aims to respond to the following research questions: –

(1) Are gender and regional inequalities in active ageing interlinked?
(2) How have regional and gender differentials in active ageing, and their combination, evolved over time?

To answer these questions, AAI breakdowns by gender and region were estimated and analyzed for different years (2007, 2009, 2012 and 2018). A linear regression was used to describe the gender-specific patterns of change of the Index by macro-regions over the considered period. The results of our study add to the emerging literature on active ageing at the sub-national level providing further insights into the diversity of regional outcomes and trends in active ageing by gender in Italy.

## 3. Materials and Methods

### 3.1. The AAI

The AAI was developed and launched in 2012 under the leadership of the United Nations Economic Commission for Europe (UNECE) and the European Commission (EC), within the framework of the 2012 European Year for Active Ageing and Solidarity between Generations. The index has been estimated periodically for all European countries, the most recent UNECE estimates being available for 2016 [37]. Reflecting the multifaceted concept of active ageing, the AAI has a multidimensional structure consisting of 22 indicators (more detailes on the complete list of indicators and on the data sources used can be found here: https://unece.org/population/active-ageing-index, accessed on 13 December 2021) grouped into four domains: employment; participation in society; independent, healthy and secure living; capacity and enabling environment for active ageing. The first three domains measure achievements towards active ageing, while the fourth provides a measure of preparedness for achieving positive result [38]. Domain-specific scores are obtained as the arithmetic weighted average of the indicators included in the domains. Similarly, the overall score is calculated as the arithmetic weighted average of the domain-specific scores. One critical aspect of the methodology consists, therefore, in the choices related to weighting. In absence of an unequivocal theoretical and empirical grounding on the contribution of each indicator to a certain domain and of each domain to active ageing, it was decided to use weights based on recommendations by an Expert Group. The AAI scores from 0 to 100, where higher scores indicate a greater extent of realizing active ageing [39].

### 3.2. AAI Breakdowns

The estimates, calculated separately for men and women for four different time points (2007, 2009, 2012 and 2018), were built using data from the national surveys (in addition to mortality tables) conducted by the National Institute of Statistics (ISTAT): Labour Force Survey (LFS), Statistics on Income and Living Conditions (SILC), Aspects of Daily Life (ADL), Family and Social Subjects (FSS), and European Health Interview Survey (EHIS). AAI is estimated separately for each Italian region with the only exception of Aosta Valley and Piedmont for which, due to data limitation (for some sources, data for the two neighbouring areas have been merged rather than being released separately by Istat. Thus, the regional groups analyzed in this study are 19 rather than 20), estimates are shown together in this article (as PIE). The Italian AAI breakdowns by sex and region have been built following the UNECE guidelines on the calculation of the AAI on sub-national levels [40]:

The procedure consisted of the following steps:

- Review of data source and data availability for the purposes of AAI calculations disaggregated by gender. This step included the identification of alternative variables (proxies) for the AAI indicators when the original data sources were unavailable for calculation.
- Compute the indicators, making sure that they correlate positively with activity and are presented as positive coefficients.
- Calculate domain’s values by multiplying the corresponding indicators with their respective weights.
- Calculate the overall AAI value by aggregating the domains values using the respective weights.
- Perform robustness checks to verify the quality and the accuracy of the calculation and ensure its consistency with the original value of the overall AAI as calculated by UNECE.

The indicators were built with the specific goal of reducing at the highest possible extent discrepancies of the Italian regional Index from the original UNECE Active Ageing Index [40]. 13 out of 22 indicators were built relying upon the same information and data sources used for the UNECE AAI (i.e., SILC, LFS and mortality tables). For the remaining 9 indicators, information was derived from the following Istat surveys: FSS, EHIS, ADL. Detailed methodological explanations concerning the data sources used for the construction of each indicator can be found in the technical report prepared by INRCA IRCSS [41] (the report also presents a comparison of values of the AAI obtained in this study with those obtained by two previous studies for Italy [34,35,36], together with a detailed discussion of the differences and similarities in the data sources used and of the potential consequences on the estimates).

### 3.3. Statistical Analyses

We rely on AAI breakdowns for year 2007, 2009, 2012 and 2018 to analyze the recent trends in active ageing of men and women across the territory. First, we provide descriptive statistics on the changes occurred between 2007 and 2018 and measure the gender gap in active ageing (calculated as the difference between the AAI value recorded by women and men) across regions and over time. Then, we use regression analysis to describe the geographical and sex-specific patterns of change in the AAI over the period considered. In particular, a linear regression is used to fit the values of the AAI (dependent variable) by year (independent variable).

## 4. Results

**Figure 1:**
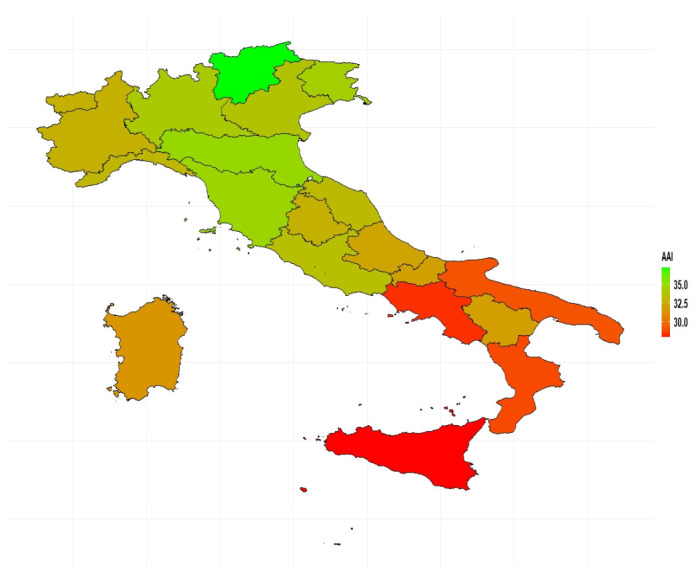
AAI by region. Year 2018. Source: Authors’ calculations based on numerous data sources from ISTAT.

**Figure 2:**
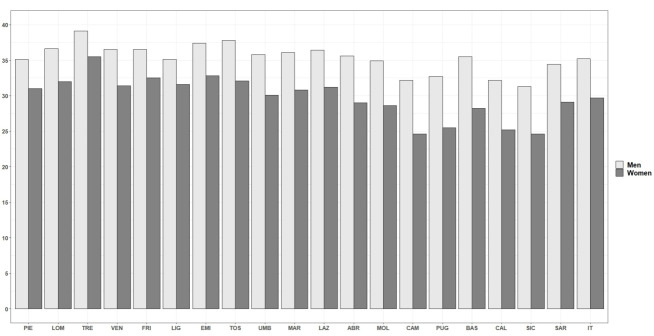
AAI by region and sex. Year 2018. Source: Authors’ calculations based on numerous data sources from ISTAT. Note: ABR–Abruzzo; BAS–Basilicata; CAL–Calabria; CAM–Campania; EMI–Emilia-Romagna; FRI–Friuli-Venezia Giulia: LAZ–Latium; LIG–Liguria; LOM–Lombardy; MAR–Marche; MOL–Molise; PIE–Piedmont and Aosta Valley; PUG–Apulia; SAR–Sardinia; SIC–Sicily; TOS–Tuscany; TRE–Trentino-Alto Adige; UMB–Umbria; VEN–Veneto.

In 2018, the AAI scored 32.4 for Italy as a whole. The overall value (i.e., for both genders combined) ranged from 27.9 in Sicily to 37.2 in Trentino-Alto Adige, thus, confirming the existence of significant regional disparities. Indeed, the AAI showed a clear geographical pattern: the values were much lower in the islands and in the southern regions than in the northern and central ones (Figure 1). A similar situation is also found when looking at the breakdowns of AAI by sex (Figure 2): in 2018, Trentino-Alto Adige recorded the highest AAI scores for both men (39.1) and women (35.5), while Sicily recorded the lowest (31.3 for men, and 24.6 for women).

**Figure 3:**
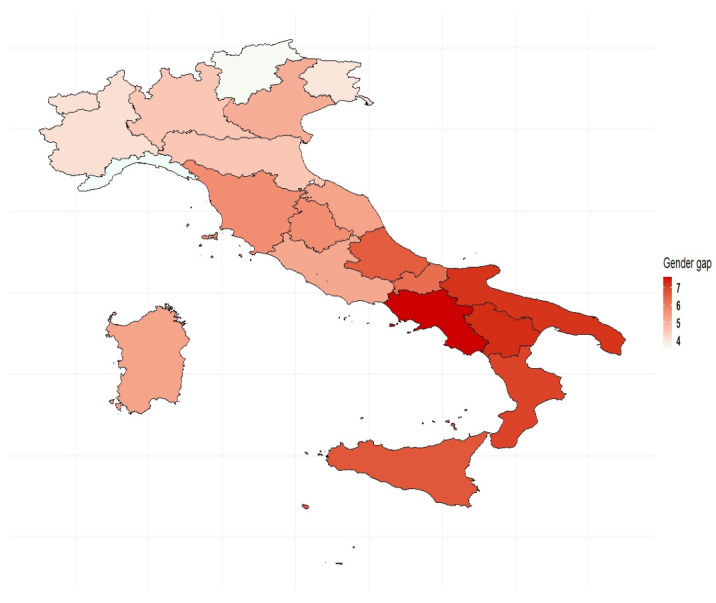
Gender gap in AAI by region. Year 2018. Authors’ calculations based on numerous data sources from ISTAT.

**Figure 4:**
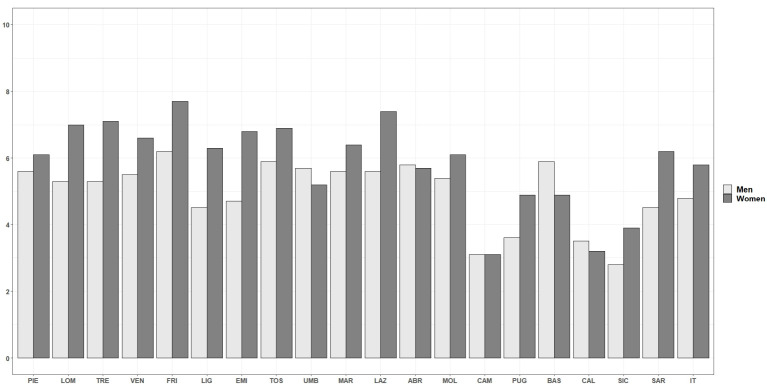
AAI by region and sex. Difference between years 2018 and 2007. Source: Authors’ calculations based on numerous data sources from ISTAT. Note: ABR–Abruzzo; BAS–Basilicata; CAL–Calabria; CAM–Campania; EMI–Emilia-Romagna; FRI–Friuli-Venezia Giulia: LAZ–Latium; LIG–Liguria; LOM–Lombardy; MAR–Marche; MOL–Molise; PIE–Piedmont and Aosta Valley; PUG–Apulia; SAR–Sardinia; SIC–Sicily; TOS–Tuscany; TRE–Trentino-Alto Adige; UMB–Umbria; VEN–Veneto.

We observe an advantage of men over women in terms of AAI score in all the Italian regions: at the national level, the gender gap was 5.6 points in 2018 and ranged from a minimum of 3.5 points in Liguria to a maximum of 7.5 in Campania. A gender gap in the AAI was recorded nationwide in 2007 and was still present in 2018 (Figure 3). In 2018, gender gaps in active ageing showed a similar (but even more pronounced) territorial gradient to that observed in Figure 1: the gender gap in AAI was greater in the southern regions than in the rest of the country. Thus, the regions with the lowest AAI scores were also those with the greatest gender inequalities. Values of the absolute changes in the AAI of men and women recorded across the country between 2007 and 2018 are shown in Figure 4. An increase in the overall value of AAI was recorded in all Italian regions between 2007 and 2018, with a gain of 5.4 points at national level (Table A1). However, this positive change was less evident in some southern regions, where the value of the absolute increase recorded between the two years fell below the Italian average. In particular, the least pronounced increases were recorded in Campania (3.2), followed by Calabria and Sicily (3.6), and Puglia (4.5). These are also the regions that showed the greatest initial disadvantage, ranking last in terms of AAI values in 2007.

Overall, regional disparities increased in 2007 compared to 2018, as indicated by the range of variation passing from 6.6 to 9.3 points (see Table A1 in the Appendix A). Looking at gender, at national level, the AAI value was 30.5 for men and 23.9 for women in 2007. The corresponding values were 35.2 and 29.7 respectively at the end of the considered period (2018), marking an advantage of women over men in terms of absolute increase with a consequent reduction of the gender gap from the initial value of 6.6 points to 5.6. Indeed, while the positive changes in the AAI between 2007 and 2018 are confirmed across the country for both sexes, women recorded higher increases than men almost everywhere. However, this progress was not enough to close the gender gap in favor of men which, as commented above, remained in all regions in 2018. Umbria, Abruzzo, Campania, Basilicata and Calabria were the only regions where the AAI increased more for men than for women. On the opposite side, the advantage of women over men was more pronounced in Trentino-Alto Adige, Emilia-Romagna and Latium than in the rest of the country. While this difference resulted from the combination of a relatively high increase for women with a relatively low increase for men in Emilia-Romagna (4.7 points, slightly below the Italian average), in Latium and Trentino-Alto Adige the difference was mainly driven by the positive changes recorded by women during the considered period (men stood above the Italian average).

**Figure 5:**
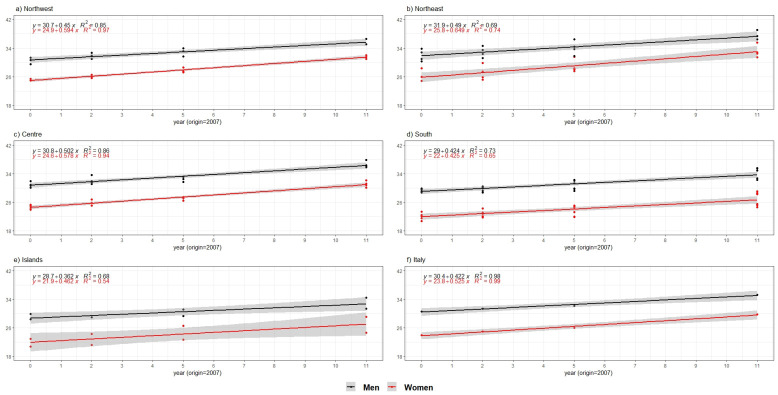
AAI trend by macro-region and sex over the 2007–2018 period. Source: Authors’ calculations based on numerous data sources from ISTAT. Note: (**a**) Northwest includes Piedmont, Aosta Valley, Liguria, Lombardy; (**b**) Northeast includes Trentino-Alto Adige, Veneto, Friuli-Venezia Giulia, Emilia-Romagna; (**c**) Centre includes Tuscany, Umbria, Marche, Latium; (**d**) South includes Abruzzo, Molise, Campania, Apulia, Basilicata, Calabria; (**e**) Islands include Sicily, Sardinia; (**f**) Italy.

The development of the indicator over the entire period is shown in Figure 5. The AAI shows a positive linear trend over time in all macro-regions of the country (i.e., Northwest, Northeast, Centre, South, Islands). At the national level, the estimated annual increase was 0.42 for men and just over half a point (0.52) for women. However, the pace of change in AAI differed across the territory: the annual increase ranged from 0.36 (Islands) to 0.50 (Centre) for men, and from 0.42 (South) to 0.65 (Northeast) for women. Statistically significant gender differences in AAI were observed throughout the period in all settings, although the results of our model suggest the existence of a gradually converging trend in active ageing over time for men and women. For instance, there were higher increases in AAI for women than for men across the country, with the only exception of the South where model estimates of the annual increase were similar for men and women. Progress in reducing the gender gap has been faster in the North and especially in the Northeast where, in 2011, women in Trentino-Alto Adige started to record AAI scores close to those of men living in the same macro-region. In contrast, it is worth mentioning that the pattern of gender convergence observed in the Islands was driven by relatively low increases in AAI recorded among men rather than by a strong acceleration of active ageing outcomes for women, as indicated by their estimated annual increase (0.462) below the national average. Therefore, our model estimates confirm evidence from the descriptive findings, indicating that regions with lower initial AAI scores and greater gender disparities are also those having made less progress in developing active ageing potential for both men and women over the period 2007–2018.

## 5. Discussion

In this article, we studied the development of active ageing in the period from 2007 to 2018 in the Italian regions focusing on differences between men and women. Results show that there has been a positive trend in active ageing in all regions in recent years. However, progress in active ageing has been unevenly distributed across the territory: increases in AAI have been lower in the southern regions and islands, which were also characterized by lower initial values, than in the rest of the country. As a result, regional inequalities in active ageing increased over time. These regional disparities in active ageing can probably be explained by the long-standing territorial dualism that still characterizes Italy [42]: Indeed, there are marked differences between the North and the South of the country in several dimensions including economic growth, social behavior and human capital [33]. Partly associated with the different availability of resources between the North and the South of the country is also “capacity”/performance of public administrations (e.g., regional governments, local authorities, health authorities) to organize and deliver services, from health and social services to waste management and local transport, as well as the provision of shared mobility [43].

In contrast, at national level, gender differences in active ageing decreased from −6.4 points in 2007 to −5.5 points in 2018. For instance, women recorded greater gains in active ageing during the period in all parts of Italy, with few exceptions. However, the amount of progress made by women has not been sufficient to close the gender gap. A gender gap in active ageing has been observed in several countries [37]. Indeed, ageing is a highly gendered process: gender differences in life experiences contribute to determining its outcomes at both individual and societal levels [12]. In particular, the traditional asymmetry in care workload has consequences in many different aspects of life (including mental health, labour market participation, financial stress, time availability) that, in turn, are likely to impact on ageing experiences. This situation is likely to be exacerbated in the future by the increasing demand for care, including long-term care, resulting from longer lives and the increasing proportions of older people in the population [44]. However, gender is not yet sufficiently incorporated into active ageing strategies, so the adoption of a life course approach to gender and ageing, which involves the structural integration of age and gender at all levels of governance, is necessary to improve current and future opportunities for labor market participation, social inclusion and active citizenship [45]. This is of fundamental relevance for Italy characterized by a strongly *familistic* welfare model, or *familialism by default* [27], that entails an almost complete shift of intergenerational care responsibilities to families (namely women) that goes along with minimal or absent policy and financial support from the state.

Our results also show that the magnitude of the gender gap was greatest in Southern Italy and the Islands, indicating that the regions that reported the lowest AAI scores were also those that reported the greatest gender inequalities. Moreover, model estimates indicate that progress in reducing the gender gap was faster in the North (and especially in the Northeast.), where initial differences between men and women were also lower, compared to the rest of the country. Previous research has shown that there is a positive association between AAI at the national level and the equality of its distribution among individuals [46]. Our findings seem to suggest the existence of a similar relationship also in relation to AAI at the regional level and the equality of its distribution across individuals of different sex, in Italy.

## 6. Conclusions

Several factors, such as migration or family policies, can influence the future of the European population, mitigating or exacerbating the ageing process; while substantial increases in old-age dependency ratios are often presented as a ‘nightmare scenario’, none of these possible future scenarios are necessarily negative, provided that population decline and ageing are properly managed [47]. To this end, policymakers need to be equipped with the necessary tools to monitor and promote healthy living and well-being for all individuals at all ages and to prevent progress towards active ageing from being unevenly distributed among different population groups.

Recent policy developments have stressed the importance of strengthening action at the sub-national level to fully develop a country’s potential of active ageing [48]. Nevertheless, empirical evidence on regional differences in active ageing is still rare. A limited number of pilot studies have been carried out to measure AAI at the regional level, pointing to the importance of regional level policies to active ageing outcomes, e.g., [49]. This article adds to the emerging literature in the filed by adding new insights into the diversity of regional active ageing outcomes and trends by gender in Italy [34,35,36], indicating that the widening geographical divide is an area deserving further consideration from national and regional authorities for the development of active ageing policies. Indeed, findings highlight that regional and gender differences interact with each other reinforcing inequalities in active ageing. By showing the persistence of disparities to the disadvantage of women in values of the AAI, the results also suggest the need to further integrate the gender dimension together with the life-cycle perspective into active ageing strategies. Despite gender has long been neglected in the debate on active ageing [11], taking into account its impact is fundamental for ageing policies for several reasons. Firstly, women represent the majority of older people. Secondly, the increase in life expectancy in old age and the decline in fertility among baby-boomers and subsequent generations cause concern about: (i) the potential supply of family caregivers; (ii) the overlapping care responsibilities for both children and older family members and their and their impact on the well-being of the family and especially women (as primary care providers). The COVID-19 pandemic has exposed underlying vulnerabilities and weaknesses related to the lives and the well-being of older people in all countries. Economic problems resulting from the health emergency are likely to influence demographic dynamics and to accelerate the ageing process, as happened in past recessions [50]. Therefore, the need to develop effective active ageing strategies is now more evident than ever.

A caveat of the approach used in this study, is that the AAI cannot capture all the possible dimensions of active ageing [51]. Indeed, although the multidimensionality of active ageing has been considered in the construction of the Index, some important aspects influencing well-being in old age have not been included, such as leisure activities [52,53]. Moreover, while the Index measures the level of involvement in activities, it does not consider how older people value, or want to be engaged in these activities [51]. One possible way to address this issue is to implement participatory and co-design approaches through the involvement of both older people and stakeholders in consultation or co-decision-making processes on active ageing policies, also based on the analysis of AAI results [9]. Another limitation of this article is that gender differences in the AAI have not been decomposed into the different domains and indicators that contribute to the overall score, an aspect that needs to be further investigated in the future. However, one of the generally acknowledged strengths of the AAI is its contribution in raising awareness of the multidimensionality and complexity of active ageing among people, including policymakers [51]. Moreover, when used for comparison, both between (e.g., at the European level) and within (e.g., regional level) countries, the AAI can help to identify challenges as well as opportunities for active ageing [37]. Therefore, by offering an overview of gender and regional diversity in active ageing and of its recent evolution, this article provides an example on how the AAI can be used as a practical tool by policy makers, at various levels of governance, to monitor trend and identify policy priorities for promoting active ageing. Future research should move in the direction of further analyzing subnational diversity in active ageing outcomes by developing detailed analyses of within-country differences in AAI domains and indicators.

## Appendix A

**Table A1:** AAI estimates by gender and territory, year 2007 and 2018.

Territory	Abbreviation	Gender	AAI	
			2007	2018
**Italy**	**IT**	M	30.5	35.2
		W	23.9	29.7
Sardinia	SAR	M	29.9	34.4
		W	22.9	29.1
Sicily	SIC	M	28.4	31.3
		W	20.7	24.6
Calabria	CAL	M	28.7	32.2
		W	22.1	25.2
Basilicata	BAS	M	29.6	35.5
		W	23.3	28.2
Apulia	PUG	M	29.1	32.7
		W	20.7	25.5
Campania	CAM	M	29.0	32.2
		W	21.5	24.6
Molise	MOL	M	29.5	34.9
		W	22.4	28.6
Abruzzo	ABR	M	29.8	35.6
		W	23.3	29.0
Latium	LAZ	M	30.9	36.4
		W	23.9	31.2
Marche	MAR	M	30.6	36.1
		W	24.4	30.8
Umbria	UMB	M	30.1	35.8
		W	24.9	30.1
Tuscany	TOS	M	31.9	37.8
		W	25.3	32.1
Emilia Romagna	EMI	M	32.8	37.4
		W	26.0	32.8
Liguria	LIG	M	30.6	35.1
		W	25.4	31.6
Friuli Venezia Giulia	FRI	M	30.3	36.5
		W	24.8	32.5
Veneto	VEN	M	31.0	36.5
		W	24.8	31.4
Trentino Alto Adige	TRE	M	33.8	39.1
		W	28.3	35.5
Lombardy	LOM	M	31.3	36.6
		W	24.9	32
Piedmont and Aosta Valley	PIE	M	29.5	35.1
		W	24.9	31.0

Source: Authors’ calculations based on numerous data sources from ISTAT.
